# Editorial: How cognitive functions interact with the motor system to shape motor behavior

**DOI:** 10.3389/fnhum.2026.2005472

**Published:** 2026-10-19

**Authors:** Antonio Parziale, Cristiano Maria Verrelli, Vignayanandam Ravindernath Muddapu

**Affiliations:** 1 Department of Information Engineering, Electrical Engineering and Applied Mathematics, University of Salerno, Fisciano, Italy; 2 Electronic Engineering Department, University of Rome Tor Vergata, Rome, Italy; 3 Multiscale Mind Lab, Psychology Group, School of Humanities and Social Sciences, Azim Premji University, Bengaluru, Karnataka, India

**Keywords:** cognitive functions, cognitive system, cognitive-motor integration, human movement, motor behavior, motor system

## Introduction

Cognitive processes such as attention, perception, memory, and decision-making critically shape motor behavior. Understanding how cognitive and motor systems interact with each other is essential for unraveling the complexities of human movement and is vital for applications spanning clinical rehabilitation, sports performance, and robotics. Historically studied in isolation, these systems are now recognized to operate in a highly integrated, reciprocal manner. Because of that, the present Research Topic has explored the complex relationship of cognitive-motor integration, investigating both the fundamental mechanisms and the applied perspectives across different contexts and populations. The contributing articles are organized into five key thematic areas (KTAs) that define such a heterogeneous field.

## KTA 1: Lifespan theories

A comprehensive approach to motor-cognitive interactions requires theoretical and evolutionary frameworks.

Klotzbier and Schott (2025) present a revised, comprehensive framework – called SMART COMPASS (Scaffolding Theory of Maturation, Cognition, Motor Performance and Motor Skill Acquisition) – that explains how genetic predispositions and environmental influences jointly shape cognitive and motor development across the lifespan. The proposed framework incorporates a compensatory scaffolding mechanism defined by the active construction and reorganization of neural structures, thereby sustaining performance despite age-related neural decline or pathological alterations.

The framework also underscores how exercise, self-efficacy, and autonomy-supportive environments may promote development, adaptation, and resilience throughout life.

At the evolutionary level, Lipp (2025) hypothesizes that what he describes as functionally over-dominant language circuits (audiomotor networks) may constrain non-verbal cognitive abilities and executive functions, including visuomotor planning and parallel processing. Lipp further proposes that the organization of the motor cortex into manual and vocal/orofacial systems may reflect a broader functional balance between visuomotor and language-related networks with a tension between “talking” and “doing”.

By further bridging neuroscience and biomechanics, Assila et al. (2026) interpret the Kinematic Theory of Rapid Human Movement as an optimal control theory. By investigating the properties of the lognormal model, they demonstrate that a composite cost function balancing movement kinetic energy, jerk, and movement duration can account for the temporal organization of rapid movements. This mathematical framework indicates that the temporal organization of rapid movements captures the internal model of limb dynamics, allowing lifelike velocity profiles to be predicted using solely kinematic controls.

## KTA 2: Action perception and prediction across the lifespan

Efficient social interaction and environmental navigation depend on the human ability to perceive, interpret, and predict others' movements. Within this setting, Ravizzotti et al. (2025) investigate how normal aging influences action prediction and intention understanding. The authors evaluate how younger and older adults predict the outcomes of everyday actions shown in videos, where action kinematics are embedded within either congruent or incongruent contextual scenarios. Their findings indicate that while older adults exhibit lower sensitivity when predicting actions interrupted early in the movement phase, they successfully compensate by relying more heavily on congruent contextual cues, which in turn enhances their response speed.

Koriakina et al. (2026) study biological motion perception by validating a novel, naturalistic video stimulus set. The authors show that neural synchrony across viewers varies as a function of which body part is moving, how the action is presented, and whether the action involves object interaction. By analyzing electroencephalography (EEG) data via inter-subject correlation (ISC) and intra-subject correlation (IaSC), they show that lower-limb movements elicit significantly stronger neural synchrony across observers than upper-limb movements or control natural scenes. The results suggest that shared neural responses are modulated by contextual features of the observed action rather than by reflecting movement alone.

Carrasco-González et al. (2026) examine the cognitive, emotional, and motor effects of Active Action Observation (AAO), a rehabilitation strategy combining intentional observation with immediate imitation. In adolescents with juvenile idiopathic arthritis (JIA), AAO is associated with a reduction in pain interference and stress, together with increased self-efficacy and reduced fear of pain and movement. This preliminary study shows the feasibility and acceptability of home-based Active Action Observation delivered through telerehabilitation.

## KTA 3: Sensorimotor predictions, agency, and expectations

A core mechanism of cognitive-motor integration involves comparing internally generated sensory predictions with actual sensory feedback. Nobusako et al. (2025) investigate this in post-stroke patients with limb apraxia. The authors identify a dissociation between impaired sensorimotor integration and preserved explicit sense of agency (SoA). Apraxic patients show a reduced ability to detect temporal mismatches between their active movements and visual feedback, suggesting impaired sensorimotor integration. However, their explicit judgment of being the agents of the resulting event remains intact. This suggests that explicit agency judgment may be preserved by compensatory cognitive mechanisms despite impaired sensorimotor integration.

To characterize the neural correlates of sensorimotor processing, Suemitsu and Nambu (2026) examine how EEG signatures during passive wrist movements induced by electrical muscle stimulation (EMS) are modulated by motor error, stimulus-driven attention, and motor imagery. Their findings suggest that deviations from internally generated sensorimotor predictions and unexpected external stimuli engage partially distinct neural responses, with late EEG activity reflecting the evaluation of motor error and earlier responses being more closely related to stimulus-driven attention.

Furthermore, expectations about our physical performance can affect human movement. Burgos-Tirado et al. (2025) present a scoping review of the nocebo effect on motor performance by examining studies in which negative expectations have been deliberately induced through verbal suggestions or conditioning. The authors show that in half of the studies considered, negative expectations affect gross motor performance, including strength, endurance, and postural stability. The review also highlights possible changes in measures of muscle activity and joint coordination, although these findings remain heterogeneous. Overall, the findings support the view that cognitive and emotional states can influence motor control.

## KTA 4: Attention, postural control, and executive demands

Postural stability demands the continuous allocation of central cognitive resources, particularly executive function and attention. Yazici-Mutlu et al. (2026) investigate the impact of sustained attention performance on postural stability in young adults with potential ADHD traits (pADHDt). They find that under cognitively demanding dual-task conditions, such as verbal backward counting, individuals with pADHDt exhibit larger center of pressure sway areas compared to controls in different stance conditions. These findings highlight the role of attentional control in balance-related motor behavior.

Similarly, Wang et al. (2026) examine whether domain-specific cognitive functions, including executive function, attention, visuospatial ability, and visual memory, are differentially associated with gait performance and postural control in acute and subacute stroke survivors. Better executive function is independently associated with faster postural movement velocity and greater maximum excursions, whereas better visual memory is associated with slower movement velocity. These findings suggest that specific cognitive domains may differentially contribute to postural control, highlighting the role of higher-order cognitive processes in the planning, monitoring, and adaptation of postural behavior.

Physical activity levels also appear to play an important role in modulating executive control processes. In this context, Liu et al. (2025) provide electrophysiological evidence that higher levels of daily physical activity are associated with distinct neural correlates of inhibitory control. Adults with higher physical activity levels show enhanced conflict-related P300 amplitudes and stronger frontal theta activity during a modified Flanker task, suggesting increased recruitment of neural resources dedicated to attention and conflict monitoring.

## KTA 5: Gait harmony, pathology, and rehabilitation

The organization of gait reflects a complex interplay between mathematical regularities, motor control, and cognitive processes. Verrelli et al. (2025) provide a mathematical framework to explain the hidden, self-similar Fibonacci patterns and golden-ratio proportions observed in cyclic human movements, such as walking of healthy subjects and tennis movements of top-level tennis players. They propose that these recursive temporal patterns may contribute to reducing information-processing demands, energy expenditure, and cognitive effort, allowing movements with many degrees of freedom to become increasingly automated.

In neurological disorders such as Parkinson's disease (PD), gait automaticity is impaired by basal ganglia dysfunction, increasing reliance on cognitive-attentional resources for locomotor control. Zhou et al. (2025) investigate the relationship between dual-task walking and specific subdomains of executive function in people with PD. Their stepwise regression analysis identifies inhibitory control, assessed using the Stroop Color and Word Test, as the strongest cognitive predictor of walking performance under both cognitive and motor dual-task conditions. These findings suggest that inhibitory control may play a key role in maintaining gait performance when walking competes with an additional cognitive or motor demand.

Cocco et al. (2025) analyze the relationship between gait alterations and cognitive deterioration with the Fibonacci mathematical framework. They show that gait harmonicity, quantified through the latest ϕ-bonacci gait number, is strongly correlated with Timed Up and Go Dual-Task (TUG-DT) performance in individuals with PD. A mediation analysis confirms that global cognition (MoCA) indirectly modulates gait harmonicity through TUG-DT, supporting the view that walking in Parkinson's disease is cognitive-dependent rather than purely automatic.

Finally, Cocco et al. (2026) provide longitudinal evidence for the sensitivity of these metrics to changes following rehabilitation. After one month of conventional multidisciplinary outpatient rehabilitation, people with PD exhibit significant improvements in functional mobility and spatiotemporal gait parameters, together with a reduction in the ϕ-bonacci gait number. Improvements in gait harmonicity are significantly associated with both clinical motor improvement and better dual-task performance, whereas global cognitive scores remain stable.

## Conclusion and future directions

The articles collected in this Research Topic illustrate that the motor and cognitive systems are fundamentally interdependent. They underscore that motor behavior is not merely an execution of physical commands, but a dynamic, top-down process heavily modulated by cognitive state, lifespan maturation, and neurophysiological constraints. The collected studies bridge the gap between computational motor control theory and empirical clinical observations in stroke, Parkinson's disease, and ADHD. Ultimately, these findings reveal that optimizing motor behavior—whether in healthy aging, sports performance, or clinical rehabilitation—requires a holistic approach that targets the underlying cognitive-motor synergies rather than treating movement mechanics in isolation. As far as the clinical practice is concerned, the findings emphasize the need to move away from isolated motor training toward integrated cognitive-motor interventions. Multimodal rehabilitation programs that combine physical exercise with cognitive challenges offer a highly effective path to enhance functional mobility, and prevent falls in aging and pathological populations. Future research should focus on validating these findings in larger clinical cohorts, and leveraging digital technologies to deliver personalized, adaptive, and ecologically valid cognitive-motor therapies.

